# Genetic variability of Hepatitis C Virus in Moroccan population

**DOI:** 10.1186/1742-4690-9-S1-P50

**Published:** 2012-05-25

**Authors:** Ikram Brahim, Abdelah Akil, El Mostafa Mtairag, Régis Pouillot, Abdelouhad El Malki, Richard Njouom, Pascal Pineau, Sayeh Ezzikouri, Soumaya Benjelloun, Salwa Nadir, Rhimou Alaoui

**Affiliations:** 1 Faculty of Sciences Ain Chock Casablanca, Casablanca, Morocco

## 

Hepatitis C virus (HCV) evolution is a highly dynamic process. There is little information about molecular epidemiology of HCV isolates in Morocco, an area known for an intermediate prevalence of HCV infection.

The primary aim of this study was to determine the subgenotype distribution of HCV strains in patients with chronic HCV infection in Morocco and an eventual association between HCV subgenotypes and liver cancer. The secondary aim was to estimate the prevalence of amino acid substitutions in the HCV core region in treatment-naive patients from Morocco and an eventual association between amino acid substitutions and liver cancer.

Serum samples from a total of 185 anti-HCV positive patients were included in this study (81 males and 104 females). The identification of HCV genotype and subtype was respectively performed by sequencing of the 5’UTR and core regions and phylogenetic analysis of the NS5B region. HCV demographic history was inferred using a Bayesian Monte Carlo Markov chain analysis. Of the 174 patients with detectable viremia, the core and the NS5B regions were amplified in 152 (87.4%) and 141 (81.0%) patients respectively. Phylogenetic analysis based on NS5B region revealed that most HCV strains were classified into subtypes 1b (75.2%) followed by subtypes 2i (19.1%), 2k (2.8%). Subtypes 2a, 1a, and 4a were found in a single patient. HCV subtype 1b had an even higher prevalence in liver cancer cases (84.4% vs 67.5% in chronic hepatitis, P= 0.031). Using a Bayesian approach, the mean date of appearance of the most recent common ancestor was estimated to be 1910 for HCV-1b and 1854 for HCV-2i. Based on core region, mutations at R70Q or L91M were detected in more than one fourth of patients infected with HCV 1b.

